# Influence of landscape management practices on urban greenhouse gas budgets

**DOI:** 10.1186/s13021-020-00160-5

**Published:** 2021-01-07

**Authors:** Wiley J. Hundertmark, Marissa Lee, Ian A. Smith, Ashley H. Y. Bang, Vivien Chen, Conor K. Gately, Pamela H. Templer, Lucy R. Hutyra

**Affiliations:** 1 Department of Earth and Environment, Boston University, Boston, MA 02215 USA; 2 Department of Earth, Environmental, and Planetary Sciences, Brown University, Providence, RI 02912 USA; 3 Department of Biology, Boston University, Boston, MA 02215 USA

**Keywords:** Climate action plan, Urban carbon cycling, Landscaping, Biogenic fluxes, Soil respiration, Nature-based solution

## Abstract

**Background:**

With a lack of United States federal policy to address climate change, cities, the private sector, and universities have shouldered much of the work to reduce carbon dioxide (CO_2_) and other greenhouse gas emissions. This study aims to determine how landcover characteristics influence the amount of carbon (C) sequestered and respired via biological processes, evaluating the role of land management on the overall C budget of an urban university. Boston University published a comprehensive Climate Action Plan in 2017 with the goal of achieving C neutrality by 2040. In this study, we digitized and discretized each of Boston University’s three urban campuses into landcover types, with C sequestration and respiration rates measured and scaled to provide a University-wide estimate of biogenic C fluxes within the broader context of total University emissions.

**Results:**

Each of Boston University’s three highly urban campuses were net sources of biogenic C to the atmosphere. While trees were estimated to sequester 0.6 ± 0.2 kg C m^−2^ canopy cover year^−1^, mulch and lawn areas in 2018 emitted C at rates of 1.7 ± 0.4 kg C m^−2^ year^−1^ and 1.4 ± 0.4 kg C m^−2^ year^−1^, respectively. C uptake by tree canopy cover, which can spatially overlap lawn and mulched landcovers, was not large enough to offset biogenic emissions. The proportion of biogenic emissions to Scope 1 anthropogenic emissions on each campus varied from 0.5% to 2%, and depended primarily on the total anthropogenic emissions on each campus.

**Conclusions:**

Our study quantifies the role of urban landcover in local C budgets, offering insights on how landscaping management strategies—such as decreasing mulch application rates and expanding tree canopy extent—can assist universities in minimizing biogenic C emissions and even potentially creating a small biogenic C sink. Although biogenic C fluxes represent a small fraction of overall anthropogenic emissions on urban university campuses, these biogenic fluxes are under active management by the university and should be included in climate action plans.

## Background

With a lack of United States federal policy to address climate change, cities, the private sector, and universities have shouldered much of the work to reduce carbon dioxide (CO_2_) and other greenhouse gas emissions. These cities—including their colleges, corporations, and faith groups—represent 155 million people [1]. While CO_2_ emissions from fossil fuel combustion have been a focus for local climate action plans, biogenic fluxes and the broader carbon (C) cycle in urban ecosystems have received less attention [2]. Many urban greenhouse gas models ignore urban biogenic fluxes due to the misconception that urban areas have negligible amounts of vegetation and soil [3, 4]. Recently, an increasing number of studies have evaluated biogenic fluxes and their contribution to C storage in ecosystems by considering spatially heterogeneous vegetation [5] and variability in soil management practices [6].

Explicitly accounting for biogenic fluxes in urban ecosystems is critical for quantifying urban C fluxes [7], as ignoring biological contributions can impact the accuracy of CO_2_ measurements via atmospheric monitoring [8] and overall reporting of total CO_2_ fluxes in urban ecosystems [4]. Climate action plans tend to focus on emissions levers like transportation policies aimed at reducing automobile dependence and increasing mass transit ridership [9]. Purchasing renewable energy and setting regulations to improve building energy efficiency as a means of reducing greenhouse gas emissions are also common mechanisms of climate action plans [10]. However, it is critical to understand how green infrastructure choices might create nature-based solutions to offset CO_2_ emissions [11] or whether they represent an unquantified additional source of emissions due to landscape management choices.

On a smaller scale, many institutions—particularly universities—have developed sustainability plans to leverage their resources and expertise to reduce their net greenhouse gas emissions, influencing the CO_2_ reduction goals of their respective cities. Boston University has pledged to achieve C neutrality by 2040, ten years before the City of Boston’s target year of 2050, and has a comprehensive plan to reduce CO_2_ emissions through renewable energy and increased energy efficiency [12]*.* Similar to other institutional actors, Boston University included C sequestration in non-urban tracts of forest that are not on the campus, but owned by the University, in their climate action plan. However, design and management of green infrastructure on urban campuses themselves may help to mitigate some of the fossil fuel fluxes and to potentially reduce energy demand via canopy shading and wind breaks [13–15]. However, mitigation of fossil fuel emissions using green infrastructure was not explicitly included in Boston University’s climate action plan, and consequently existing nature-based solutions were not considered in this study. While food waste, human respiration, and many other factors also influence the biogenic C balance [16], this paper focuses on biogenic C fluxes in plants and soils of urban ecosystems across three highly urban campuses owned by Boston University.

Ecosystem C exchange includes uptake of C through photosynthesis and losses through respiration, with the difference between these two processes resulting in either a biological sink (uptake) or source (release) of atmospheric CO_2_. Soil respiration contributes about 70% of respiratory losses in CO_2_ in temperate forests [17], while CO_2_ fluxes from soil respiration in lawn and landscaped landcover in urban areas can be the same order of magnitude as fossil fuel emissions, especially in intensively landscaped environments such as suburbs [6]. Management choices to amend the soil (e.g. addition of compost and mulch) can more than double CO_2_ respiration rates relative to nearby rural forests [6]. Increased rates of soil respiration due to mulch application, for example, mean that landscaping “beautification” efforts can have significant effects on the rate and magnitude of C emitted from soils. Landscaping maintenance activities also have direct fossil fuel emissions associated with activities such as crew and product transportation, lawn mowing, leaf blowing, and fertilizer application [18], as well as the operation of bucket trucks, chainsaws, and wood chippers during tree maintenance [19]. Landcover management decisions in urban areas therefore have the potential to greatly influence the direction and magnitude of biogenic C cycling in urban ecosystems.

Similar to soil respiration, C sequestration in trees can be greater in urban ecosystems compared to adjacent rural areas. Briber et al. [20] found that urban tree growth rates accelerate up to twice those in rural forests following urbanization, suggesting that CO_2_ uptake in urban trees plays a significant role in the urban C cycle. However, the shorter lifespan of urban trees compared to rural trees can affect the ecosystem’s ability to effectively store C on a large scale [21].

Few studies have quantified C fluxes through urban ecosystems using bottom-up estimates of landcover type and flux rate. Hardiman et al. [4] estimated biogenic C fluxes for Massachusetts using a 30 m resolution biomass map coupled with the Vegetation Photosynthesis and Respiration Model. Other bottom-up approaches assessed the effects of human presence on soil C content [22] and compared estimates of emissions from energy consumption to local biogenic uptake measurements [23]. Additional modeling approaches have used established remote sensing methods—such as Light Detection and Ranging (LiDAR) and multi- and hyperspectral images—to map urban forest structure and estimate C storage [3, 24, 25].

Our study uses in situ measurements and meter-scale landcover classification data to quantify C fluxes in terrestrial ecosystems on Boston University’s urban campuses to assess the role of biogenic C fluxes within the broader C budget. We focused primarily on C loss from mulch and lawn respiration and net C uptake by trees and grass, as well as anthropogenic CO_2_ emissions associated with heating and electricity (referred to as Scope 1 and Scope 2 emissions, respectively). This study fills an important gap in knowledge of C management research, as urban areas historically have not been major focal points for ecologists examining the role of biogenic fluxes in the C cycle [26].

## Materials and methods

### Site description

The greater Boston area is the most populous metropolitan area in Massachusetts and the tenth largest metropolitan area in the United States [27]. Boston has a temperate climate, with mean winter and summer temperatures of − 0.1 °C and 21.7 °C, respectively; mean annual precipitation is approximately 1112 mm [28]. Boston University owns land on five campuses: Charles River, Medical, Wheelock, Sargent Center, and Tanglewood Campuses. Boston University’s main campus, the Charles River Campus (74 ha), lies parallel to the Charles River in the City of Boston (Additional File 1). Boston University owns two additional urban campuses in Boston; the Medical Campus (32 ha) in the South End and the Wheelock Campus (3.2 ha) in the Fenway neighborhood of Boston (Additional File 1). Outside of Boston, the University owns the Sargent Center (302 ha), a conference center and Nature’s Classroom in Hancock, New Hampshire, and the Tanglewood Campus (26 ha), home to the Boston University Tanglewood Institute in Lenox, Massachusetts, both of which are rural.

In early June 2017, ten field plots ranging from 179 to 3963 m^2^ (Table 1) were established on the Charles River Campus. Plot locations were selected based on patch sizes and to be representative of the two dominant vegetated landcover types present on the three urban Boston University campuses: lawn and mulched landscapes. Each plot was dominated by ornamental, non-native hardwood trees common to northeastern United States cities. All trees were intentionally planted and are actively managed by the University landscaping crew.

**Table 1:** Areas of plots on the Boston University campus. Number of trees with dendrometer bands and number of soil collars are listed for each plot in 2017, with the number of samples for 2018 in parentheses

Plot number	Area (m^2^)	Cover types included	Number of trees with dendrometer bands	Number of soil collars
1	1427.09	Lawn, mulch	8 (3)	2 (1)
2	870.11	Lawn	8 (7)	2 (2)
3	179.25	Mulch	8 (8)	2 (2)
4	389.37	Lawn, mulch	8 (8)	2 (2)
5	3101.81	Lawn	8 (0)	2 (0)
6	2175.38	Lawn, mulch	8 (8)	3 (2)
7	3962.99	Lawn, mulch	8 (8)	2 (1)
9	182.93	Mulch	4 (4)	0 (0)
11	544.36	Lawn, mulch	5 (4)	3 (3)

### Soil respiration and tree growth

We installed two or three 20.2 cm-diameter polyvinyl chloride (PVC) collars per plot in June 2017 to measure CO_2_ efflux across the two landcover types (Table 1). Plots with more than one landcover type present had one soil collar installed in each landcover type. We had a total of nine collars on lawns and nine in mulched areas. Consistent with Decina et al. [6], soil collars were left to equilibrate for 2–3 weeks in order to avoid large pulses in CO_2_ efflux from roots severed during installation. CO_2_ flux was measured using a 20-cm chamber soil CO_2_ efflux system (LiCOR-8100A) from 05 July to 31 October 2017 and 8 May 2018 to 29 November 2018, with an additional measurement made on 28 January 2019. Because effectively all landscape management occurs during the growing season (April–October), observations made across the entire growing season ensured that measurements represented a “typical” year of landscape management on Boston University’s urban campuses. During the winter months, deciduous tree growth is effectively zero and soil respiration rates are extremely low with very cold and potentially snow covered soils. Soil respiration measurements were made weekly between late morning and mid-afternoon in a randomized order across plots to account for variation across time of day.

To measure rates of tree growth, dendrometer bands were installed on hardwood trees across the ten plots (n = 73 trees total) in June 2017. Dendrometer bands were placed at 1.37 m above the ground to measure changes in tree diameter over time. Tree growth was measured monthly from 10 May through 30 November 2018. Measurements were made using digital calipers. In 2018, dendrometer band and soil flux measurements continued on eight out of ten plots, as one plot (Plot 5) was removed from sampling due to peeling tree bark and another (Plot 8) was removed entirely due to paving in part of the plot. A final total of 50 trees were measured monthly for growth.

### Landcover data processing

**Fig. 1:**
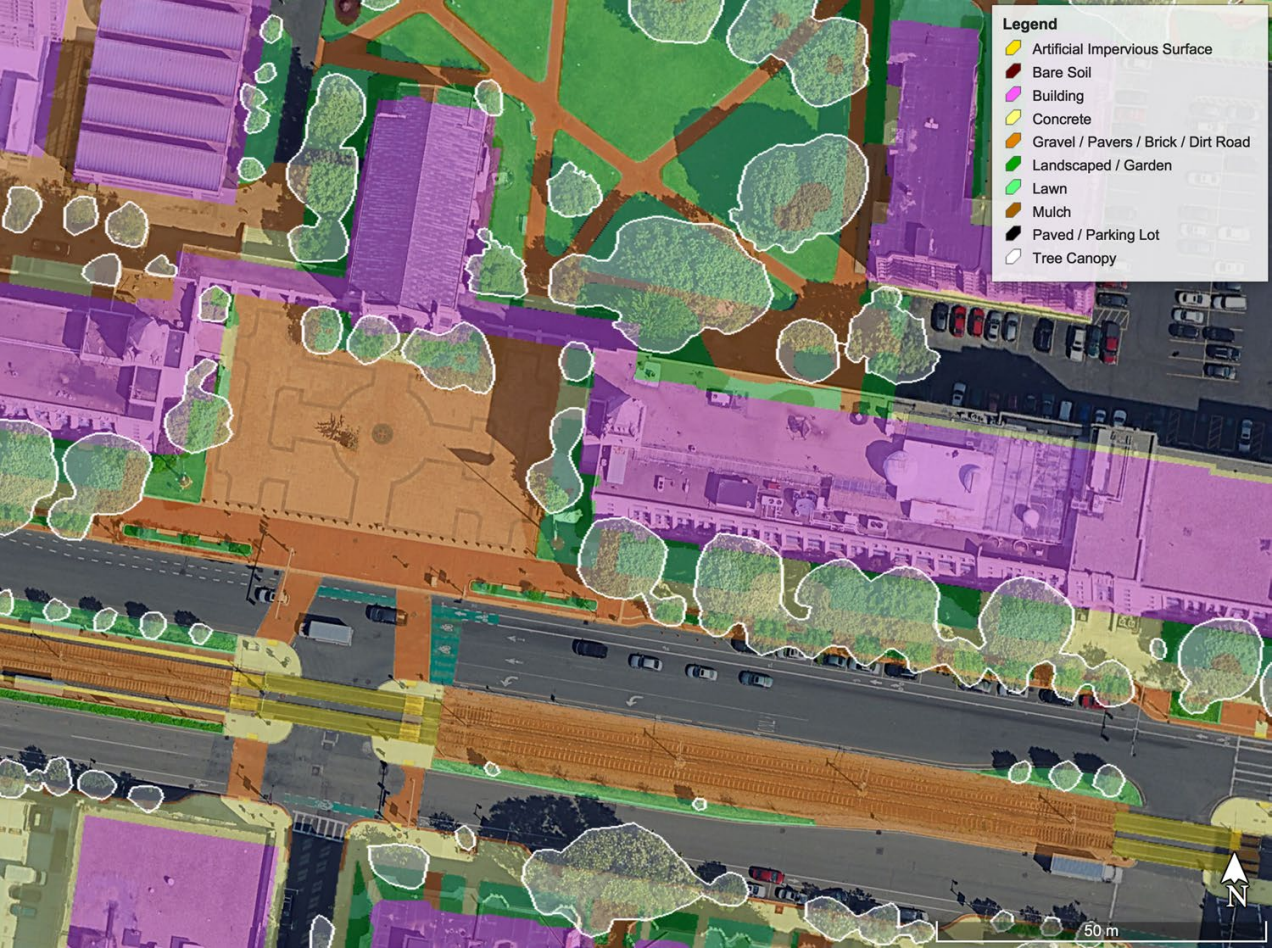
Landcover type classification on the Marsh Plaza and BU Beach areas of Boston University’s Charles River Campus

**Fig. 2:**
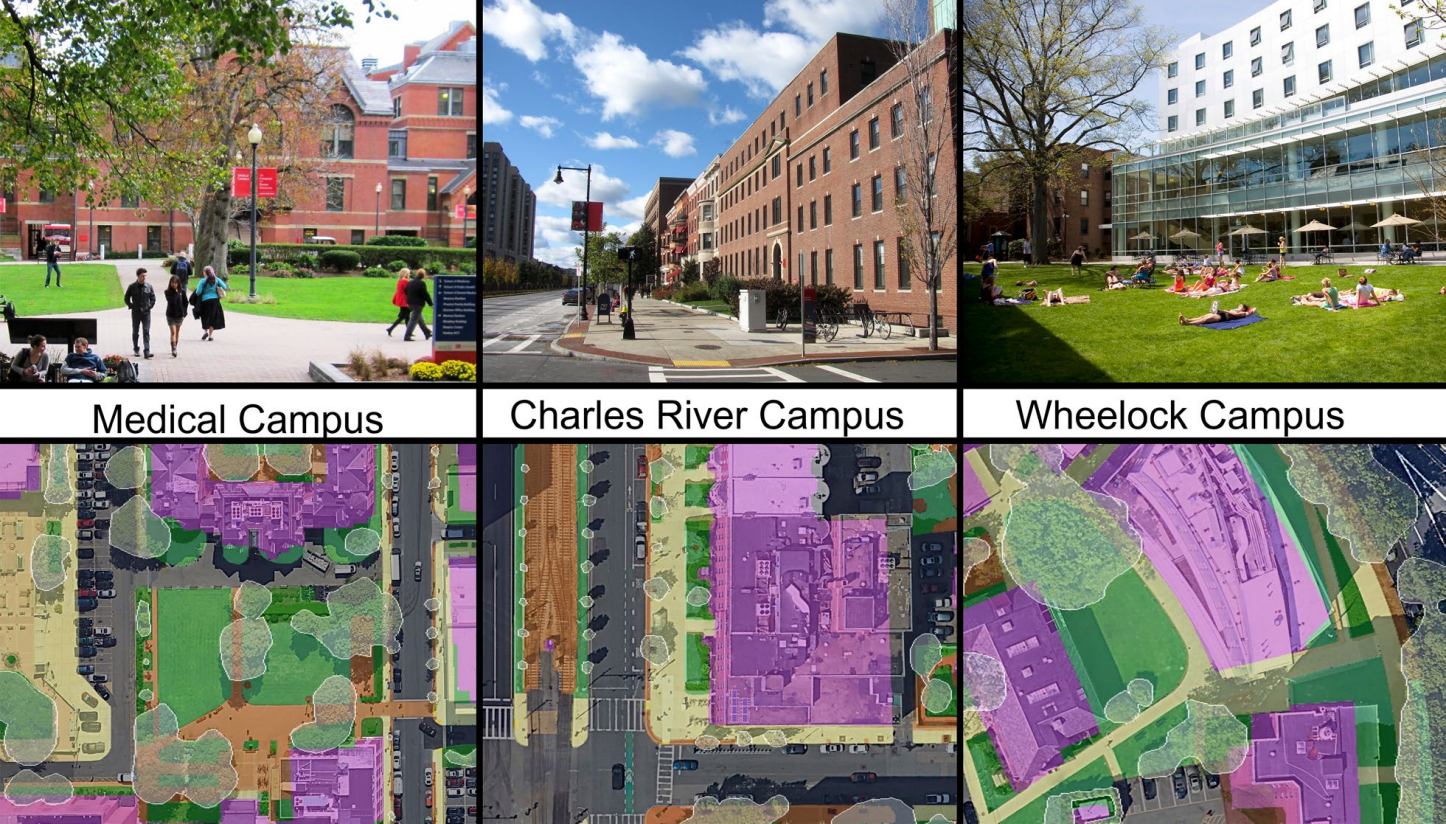
Digitization examples from each campus; the Medical Campus (32 ha), the Charles River Campus (74 ha), and the Wheelock Campus (3.2 ha). Refer to Fig. 1 for landcover types and colors. (Image Credits L to R: BU Alzheimer’s Disease Center, John Phelan, IEC Abroad)

Shapefiles with parcel boundaries were used to define the study areas across the five campuses, with the exception of the Wheelock Campus (acquired in 2018), which was manually digitized using Google Earth (Version 7.3). All parcels were projected onto the Google Earth base map, and 13 landcover classes were manually digitized with a minimum mapping unit of roughly 1 m^2^: artificial impervious surface (i.e., track, AstroTurf, rubber rail crossings), bare soil, building, concrete, forested, gravel/pavers/brick/packed dirt road, landscaped garden, lawn, mulch, parking lot, paved, water, and thinned forest (Fig. 1). An overlaying tree canopy class was created to identify and quantify where tree canopy covers the Boston University campuses (Figs. 1, 2). Google Earth satellite imagery was used primarily from April 2017 and was supplemented with imagery from June 2017 and October 2018. When possible, landcover classifications were verified using Google Earth’s Street View feature or visited in-person. Canopy cover polygons were drawn using leaf-on imagery from June 2017, although imagery from October 2018 was also used to delineate Tanglewood Campus canopy cover. The digitization process resulted in 5,560 unique polygons across all five campuses.

Using ArcGIS Pro (Version 2.0.1), each polygon was converted into a shapefile and projected onto a map using the North American Datum 1983 StatePlane Massachusetts Mainland 2001 projected coordinate system. The geodatabase produced by ArcGIS was read into R (The R Project for Statistical Computing, version 3.4.3), which was used for all remaining data processing.

### Carbon flux data processing

Soil respiration fluxes, in µmol CO_2_ m^−2^ s^−1^, were estimated using locally estimated scatterplot smoothing (LOESS) fit across observations as a function of lawn and mulch landcovers. For mulch and lawn landcover, observations from 2018 and the singular measurement from January 2019 were used. To account for uncertainties associated with human tampering and instrument stabilization, 36 of the 50 dendrometer bands were ultimately deemed reliable to include in our estimates of tree growth rates across the Charles River Campus, although one additional tree was removed from the study due to lack of end-of-season data on diameter at breast height (DBH). Tree stem and branch aboveground biomass was calculated using a generalized allometric equation for mixed hardwood species aboveground biomass:

1 $$bm = {\mathrm{Exp}}(-2.4800 + 2.4835{\mathrm{ln}} dbh)$$

where *bm* is total aboveground biomass (in kg) and *dbh* equals the DBH (in cm) [29]. The component contribution of foliage:

2 $$foliage\, ratio ={\mathrm{ Exp}}(-\,4.0813 + 5.8816/dbh)$$

was subtracted from total aboveground biomass [29]. For each of the 35 trees, the change in biomass (increment) was calculated by the difference between the initial and final biomass and divided by the total amount of time between measurements. The aboveground biomass increment over the 2018 growing season was multiplied by 0.5 to convert biomass to C mass [30]. Annual leaf production was estimated based on a locally observed ratio of woody growth to leaf production of 0.50 ± 0.01 at the nearby Harvard Forest [31]; following Trlica et al. [32] we doubled the estimate of tree C uptake to account for leaf production. Accounting for foliar C as uptake is critical because C fixed in leaves is later released during the soil respiration process. Inclusion of respiration losses without calculating foliar C uptake would result in fundamentally biased estimates.

Rates of C sequestration by turf grasses across the Charles River Campus were calculated using sequestration estimates from Kaye et al. [33], who quantified C sequestration on well-maintained urban lawns in Fort Collins, Colorado. Kaye et al. [33] determined that the C sequestered by mowed grass over the course of the growing season was 133 g C m^−2^ year^−1^, while unmowed, end-of-season grass sequestered 42 g C m^−2^ year^−1^. These values were scaled to the Boston University urban campuses using the lawn area of each of the University’s campuses. We are unaware of any analogous published studies closer to Massachusetts.

For each of the 35 trees equipped with dendrometer bands, a polygon was drawn in Google Earth to represent the tree canopy area to estimate the ground area (in m^2^) covered by each tree’s canopy. Each tree’s estimated annual C uptake (in kg year^−1^) was divided by its canopy area (in m^2^) to estimate C uptake as a function of tree canopy area across each of Boston University’s urban campuses. C uptake was estimated on the basis of both the total land area and the amount of pervious land area using the digitized landcover. Monthly growth increments of C uptake were estimated by determining monthly growth rates for each tree across the 2018 growing season. These resulting growth increment calculations were used for both growth by aboveground woody biomass as well as grass growth on campus. Monthly tree growth increments were used to allow for the breakdown of annual woody biomass growth into discretized months so monthly lawn and mulch respiration (C loss) and sequestration in trees and grass (C gain) could be compared. Additionally, utilizing in situ measurements of tree growth allowed for measurement of these monthly growth increments and increased local accuracy. Using Google Earth for polygon creation—including canopy extent—ensured that all open-source remote data were used, allowing Boston University community members to easily visualize the landcover and allowing other universities to conduct similar assessments of their landcover.

Net C exchange was calculated by subtracting the aboveground C sequestration (into woody biomass, leaves, and grass) for the year from the total soil respiration in units of Mg C year^−1^. This calculation was completed for each of the three urban campuses owned by Boston University. Positive values indicate net fluxes of C to the atmosphere, while negative values indicate net fluxes of C into vegetation.

### ***Anthropogenic CO***_***2***_*** emissions calculations***

We estimated both combined Scope 1 emissions from Boston University buildings (direct, local combustion) and Scope 2 emissions associated with electricity usage (emitted at a power plant elsewhere) for four of Boston University’s campuses in 2016. Scope 1 emissions attributable to Boston University from 2016—the most recent available year of data—were independently estimated based on the Anthropogenic Carbon Emissions System (ACES), which calculated kg of CO_2_ produced by all commercial and institutional buildings within a Census Block Group [34]. Estimates of kg CO_2_ from the ACES model were divided by the total building floor area (m^2^) within each of the block groups, obtained from the Environmental Protection Agency’s Hazus database [35], to produce the average kg CO_2_ per square meter of building space per year for each census block. This average was then multiplied by building area within each Level 3 Assessors’ parcel—obtained from MassGIS Data—on the Boston University Charles River, Medical, and Wheelock Campuses.

We also obtained a second, independent CO_2_ emissions estimate for 2016 from Boston University’s Sustainability Program, which tracks University-wide greenhouse gas emissions (Scope 1 and 2) as metric tons of carbon dioxide equivalents (MTCO_2_e) and breaks these emissions down into the Charles River Campus and the Medical Campus by the utility percentage. The University does not meter most individual buildings or even individual campuses. Emissions data for the Charles River Campus also include the Tanglewood and Wheelock Campuses, and the Medical Campus measurements are further broken down into the Medical Campus and the National Emerging Infectious Diseases Laboratories (NEIDL). Because NEIDL is on the Medical Campus, these two estimates were grouped into one integrated estimate of the emissions from the Boston University Medical Campus. For both the ACES and University-based emissions, MgCO_2_ and MgCO_2_e were converted into MgC using molecular weights. Both estimates contained annual emissions as this was the smallest temporal unit for which data was readily available.

### Statistical analyses

All statistical analyses were conducted in R. All reported error values represent 95% confidence intervals. Spatially aggregated estimates were weighted based on the amount of associated landcover characteristics; errors were calculated as a root mean square to combine errors across multiple sources. Shapiro-Wilkes Test for normality showed that soil respiration data were not normally distributed for both mulch and lawn landcover (p = 4.7 e−10, p = 7.8 e−05), and that C sequestration rates in kg C m^−2^ of canopy cover were also not normally distributed (p = 3.1 e−05). Linear mixed effects models were used to conduct t-tests using Satterthwaite's method, providing p-values in the comparison of flux rates for mulched and lawn landcover, and for comparing flux rates between years. Within each year, landcover types were set as a fixed effect with day-of-year as a random effect, and between years both landcover type and year were set as fixed effects.

## Results

**Fig. 3:**
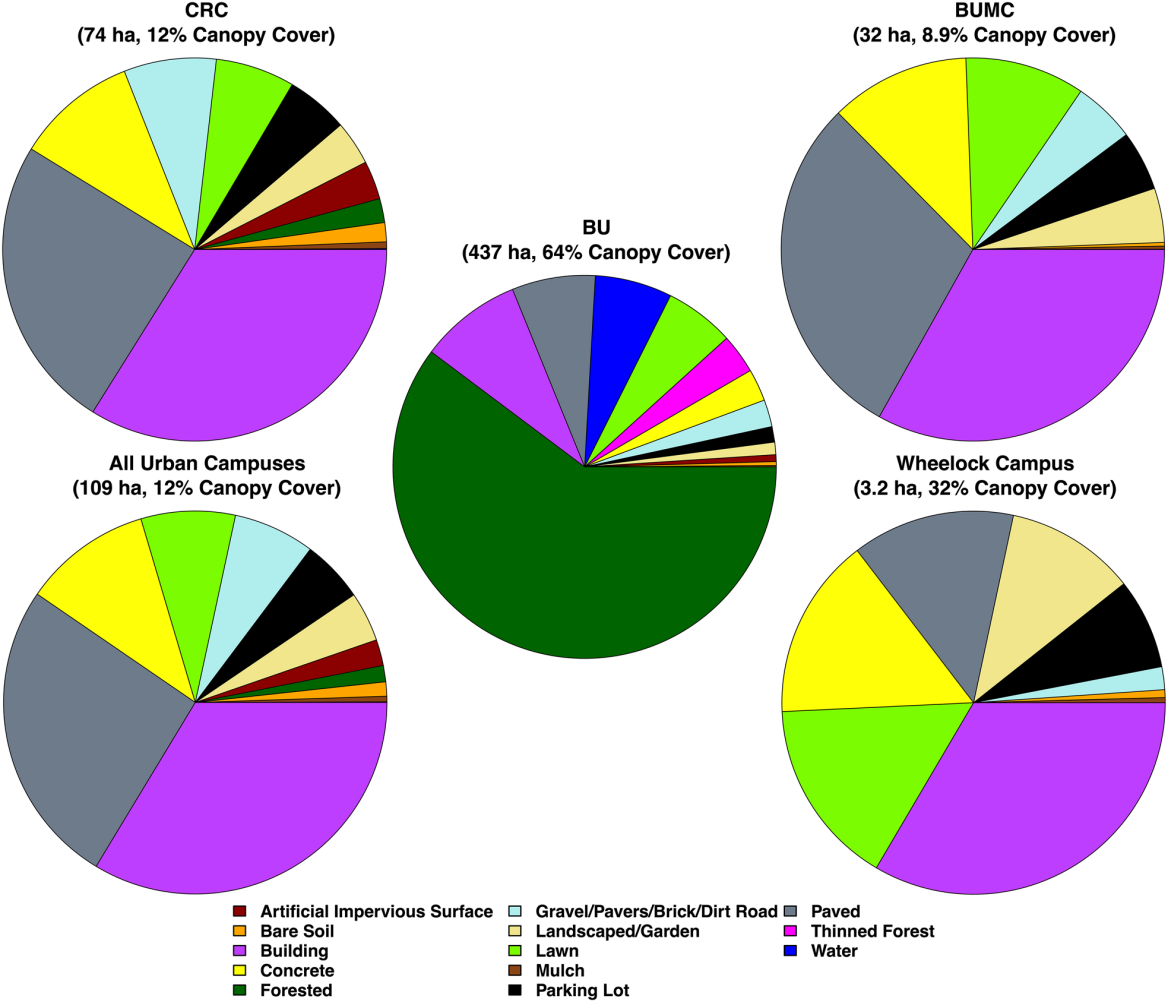
Proportional landcover for each urban campus at Boston University, as well as an average of all urban campuses. Campus canopy cover and campus areas in hectares are also listed

Across the three urban Boston campuses, paved, building, and concrete surfaces are dominant across the landscape (Table 2). The remaining landcover was composed of more permeable materials such as lawn, landscaped garden, and brick or gravel (Fig. 3). The Charles River Campus has 22.4% pervious surface area and 12.2% canopy coverage. The Wheelock Campus is less than 1 km away, but resembles a more quintessential college campus as it has proportionally more lawn and mulched areas than the other urban campuses; Wheelock Campus has 29.8% pervious surface area and 31.6% canopy coverage. By contrast, the Medical Campus is the most highly developed campus, and it includes extensive hospital infrastructure and developed landcover with 20.5% pervious cover, and 8.9% canopy coverage.

**Table 2:** Extent of lawn and mulched landcover on each of Boston University’s urban campuses, as well as total biogenic emissions and sequestration, net biogenic C flux, and ACES-derived Scope 1 emissions

Campus (area)	Mulch cover (%)	Lawn cover (%)	Emissions via mulch (Mg C ha^−1^ year^−1^)	Emissions via lawn (Mg C ha^−1^ year^−1^)	C sequestered (Mg C ha^−1^ year^−1^)	Net Biogenic C flux (Mg C ha^−1^ year^−1^)	Scope 1 emissions (Mg C ha^−1^ year^−1^)
Charles River Campus (74 ha)	4.3	6.7	0.7 ± 0.18	0.9 ± 0.25	0.9 ± 0.67	0.8 ± 0.74	248
Medical Campus (32 ha)	4.8	10.1	0.8 ± 0.21	1.4 ± 0.38	0.7 ± 0.44	1.5 ± 0.62	440
Wheelock Campus (3.2 ha)	11.4	15.8	1.9 ± 0.49	2.2 ± 0.60	2.2 ± 0.67	1.9 ± 1.02	201

**Fig. 4:**
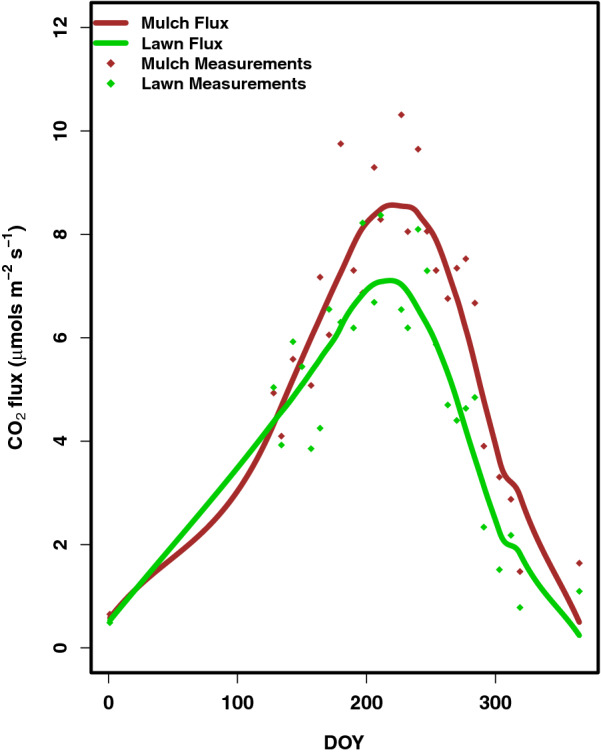
Measured flux rates of mulch and lawn landcover in 2018, and the LOESS curve used to predict flux rates year-round

**Fig. 5:**
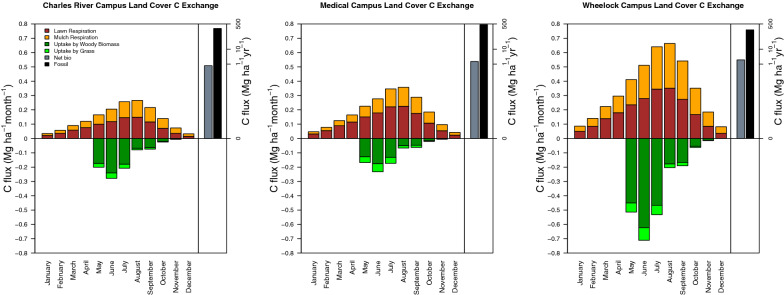
Monthly C respiration by mulch and lawn and sequestration by woody biomass and grass. Net biomass flux and anthropogenic emissions estimates are represented on a logarithmic scale. All numbers are per hectare, as they have been normalized by the unit area of the respective campus

Soil respiration rates in lawn areas averaged 5.5 ± 0.5 µmol CO_2_ m^−2^ s^−1^ and 5.0 ± 0.4 µmol CO_2_ m^−2^ s^−1^ for 2017 and 2018, respectively (Fig. 4). Respiration rates for mulch averaged 6.8 ± 0.7 and 6.3 ± 0.6 µmol CO_2_ m^−2^ s^−1^ for 2017 and 2018, respectively. Between 2017 and 2018, average flux rates were not significantly different (p = 0.35). Within both 2017 and 2018, rates of respiration in mulched landcover were significantly higher than lawn landcover (p = 0.012, p = 0.00012, respectively). Rates of respiration were greatest in the months of July and August (Fig. 5).

Aggregating soil respiration fluxes from both landcovers, we find that the Charles River Campus emitted the least biogenic C per unit area, whereas the Wheelock Campus emitted the most biogenic C per unit area (Table 2). Further, each of the campuses contained more lawn than mulched landcover, though for the Charles River Campus and the Wheelock Campus, mulched and lawn landcover added roughly the same amount of C to the overall flux of CO_2_ to the atmosphere.

Woody biomass sequestered 16.8 ± 6.9 kg C year^−1^ tree^−1^, and C sequestration rates were not statistically significant between trees planted in mulched and lawn landcovers across 2017 and 2018 (p = 0.50). C sequestered by trees was greatest in May, June, and July, and was modeled to be zero from December through April. On both the Charles River Campus and the Wheelock Campus, C loss through respiration was higher than sequestration each month of the year except for May and June, when C sequestration into woody biomass and grass outweighed C loss (Fig. 5). On the Medical Campus, C loss through respiration was higher than sequestration during each month of the year. Consequently, each campus was an annual net source of biogenic C.

Using the ACES model, we found that Scope 1 building heating related emissions on the Charles River Campus were 248 Mg C ha^−1^ year^−1^ in 2016, the Medical Campus was 440 Mg C ha^−1^ year^−1^, and the Wheelock Campus emitted 201 Mg C ha^−1^ year^−1^. University electricity usage data – which combines the Charles River, Tanglewood, and Wheelock Campuses – showed that the three campuses produced a combined 263 Mg C ha^−1^ year^−1^ and the Medical Campus emitted 258 Mg C ha^−1^ year^−1^ in 2016.

Additionally, there was significant variation in the proportion of emissions through biogenic respiration to anthropogenic emissions. On the Charles River Campus, respiration equaled 0.67% of Scope 1 emissions, whereas respiration represented 0.51% of Scope 1 emissions on the Medical Campus and 2.06% of Scope 1 emissions on the Wheelock Campus (Table 2). This calculation was repeated to find the proportion of net biogenic emissions to anthropogenic emissions.

## Discussion

Climate action plans allow cities and institutions to create an achievable framework for reducing their greenhouse gas emissions. Specifically, these plans provide targets for emissions reductions within a given time-frame and implementation pathways for reducing emissions through clean technologies, renewable energies, changes in transportation patterns, and the use of green infrastructure. Boston University owns roughly 0.76% of the combined land areas of the City of Boston and the bordering Town of Brookline, a sizeable portion of metropolitan Boston's land for a single land owner, making it particularly important that the University accurately accounts for C fluxes since these fluxes can have a significant impact on the City of Boston as a whole. Our estimates across the three Boston University campuses show that all were a net source of biogenic C to the atmosphere due to landscaping management practices and that biogenic emissions comprised 0.51% to 2.06% of the total anthropogenic emissions on each campus. This is a lower-bounds estimate as it does not include direct emissions associated with maintenance such as lawn mowing, transportation, and leaf blowing. Considering these sources, lawn mowing and fertilizer application alone increase Charles River Campus biogenic C emissions by 1.0% under typical non-irrigated “home” lawn management practices [18], with an additional 2.3 to 9.5 kg C in maintenance for each tree each year [19, 36].

Lawns comprise a higher proportion of landcover than mulch on each of the three urban campuses, although on the Charles River Campus and Wheelock Campus overall C loss was roughly equal between mulch and lawn due to the higher rate of C flux per unit area from mulched areas. The Wheelock Campus’ high rate of C respiration was attributed to its high overall pervious landcover fraction. Nearly a third of the Wheelock Campus is covered by lawn, mulch, or soil, whereas the Charles River Campus and the Medical Campus had lower overall rates of C respiration due to the prevalence of impervious surfaces (77.6% and 79.5%, respectively). However, Wheelock also had over twice the amount of canopy cover as the Charles River Campus and nearly four times that of the Medical Campus, offsetting 47.3% of respiratory emissions of CO_2_.

Both the Charles River Campus and the Medical Campus had substantially lower tree canopy cover (12.2% and 8.9%, respectively) than the City of Boston’s average of 26% [3]. Sequestration into woody biomass and grass offset 54.0%, 52.7%, and 32.7% of biogenic emissions from soil respiration for the Wheelock Campus, Charles River Campus, and Medical Campus, respectively.

Of Boston University’s urban campuses, Wheelock Campus most closely resembles a “traditional” college campus with a large, manicured grass quad surrounded by campus buildings (Fig. 2). The relatively high proportion of vegetation and canopy cover facilitates landcover modification. Over half of Wheelock Campus’s biogenic C emissions are offset by on-campus tree and grass sequestration, suggesting that smaller changes in on-campus landcover management strategies such as minimizing mulch application and increasing tree canopy cover could contribute to Wheelock achieving biogenic C neutrality or even the potential creation of a small biogenic sink.

Although transportation of goods, services, and University community members to and from campus represent a significant source of University-based anthropogenic emissions, granular emissions data from fossil fuel combustion were not available for this analysis. The Boston University Climate Action Plan focuses primarily on Scope 1 and Scope 2 emissions, and estimates that University-based indirect personal transportation, purchasing, and waste disposal emissions are approximately 200,000 MTCO_2_e (54,582 Mg C) [12]. Including Scope 1 and Scope 2 emissions, over which the University maintains greater control, allows for the most accurate estimates of University-related anthropogenic emissions and represents the emissions most easily regulated by the University.

The ACES emissions inventory is at a census block scale, including areas beyond the campus boundary. The University’s own emissions inventory aggregates across campuses since it is not sub-metered and includes both direct Scope 1 building emissions and indirect Scope 2 electricity emissions. Many large, institutional land owners often do not have sub-meters for their many buildings, making it difficult to disaggregate emissions data and assess efficiency improvements. The ACES model estimates for Scope 1 C emissions in the Medical Campus were significantly higher than the Charles River and Wheelock Campuses, likely due to the high proportion of industrial buildings—which were modeled with higher CO_2_ emission rates than residential buildings—in the Medical Campus’ census block. While the exact emissions are more uncertain than would be desirable, it is clear that Scope 1 and Scope 2 emissions far surpass biogenic emissions on each urban campus. For example, biogenic emissions from lawn and mulched landcover on the Medical Campus represent just 0.51% of Scope 1 C emissions from the Medical Campus. On the Wheelock Campus, which has considerably lower anthropogenic C emissions, biogenic emissions make up 2.06% of all C emissions.

Although a breakdown of anthropogenic emissions into smaller temporal/spatial intervals is unavailable in this study, seasonal variability is incorporated into emissions estimates from ACES and the University-based emissions inventory. ACES, for example, found that summertime anthropogenic emissions represent 57% of wintertime emissions, largely due to the lack of heating-related emissions during the summer months [6, 37]. Scheduled variations in on-campus activity—such as holiday breaks, graduation, and move-in weekend—also likely affect campus anthropogenic emissions, although the campus is heavily populated year-round, including during the intersession summer months. While these variations are not explicitly included in ACES emissions estimates, they are accounted for in the University’s emissions inventory. Variations in landscape management also occur sporadically over the course of the growing season, particularly during campus “beautification” initiatives preceding high-profile campus events such as parents’ weekend, alumni weekend, and commencement weeks. This suite of management and seasonal variations is included in our measurements of biological fluxes of soil respiration and tree growth across an entire season of landscape management. It is also worth noting that management intensity may slightly vary between campuses due to the relative prevalence of high-profile campus events on some campuses.

Considering C sinks on non-urban lands – such as Boston University’s Sargent Center and Tanglewood Campuses—affects Boston University’s entire C budget. The Sargent Center and Tanglewood Campuses, for example, include 2.76 km^2^ of forest or thinned forest, which helps to offset 4,700 Mg CO_2_ (1,283 Mg C) of the University’s C emissions [12]. This study focused on Boston University’s three urban campuses, but a University-wide assessment should include C respiration and sequestration estimates from non-urban satellite campuses to quantify University C fluxes across all lands owned by universities. The question of additionality—or the allowance of C credits for business-as-usual management of forests—needs to be considered in both urban and non-urban satellite campuses [38]. This approach might allow universities such as Boston University to claim C credits for maintaining forested land as a C sink, offsetting biogenic or anthropogenic emissions from their urban campuses. However, this C credit can only be issued under the recognition that the University maintained this sink for the purpose of C sequestration, as opposed to receiving credit for simply following a “business-as-usual” plan.

This analysis points to several important data shortfalls and opportunities for managing C fluxes. Mulched soils have a significantly higher rate of C loss from soil respiration than lawns, indicating that replacing mulch with ground cover plants or even lawn—minimizing the application of mulch on campuses—has the potential to greatly reduce C emissions from soil respiration. Preceding high-profile campus events, mulch is very liberally applied across the campus for “beautification.” Applying a thinner layer of mulch or applying biennially will assist with reducing soil respiration rates. Although C respiration rates vary based on the amount of mulch applied—as well as other maintenance methods and weather patterns—this study included growth and respiration measurements over multiple years with varying temporal frequencies to capture the timing and controls on biogenic fluxes. The observed inter-annual variability in measured C fluxes is within the range typically observed in natural systems [39].

We do note that mulch does have some environmental benefits that we have not accounted for here, particularly insulating soils to retain moisture [40]. However, most of the Boston University campus is well irrigated with timed sprinklers, reducing the need for hydrological benefits from mulch. Planting additional trees has potential to mitigate some of the high losses of CO_2_ from soil respiration on University campuses. Two of the urban Boston University campuses (i.e. the Charles River and Medical Campuses) have particularly low canopy cover compared to the City of Boston; there are opportunities to increase canopy coverage to help offset on-campus emissions and potentially reduce building energy demand through canopy shading effects [13–15, 41].

Critical components not included in this study include investigating the tradeoffs between increased canopy cover and respiration rates, as some potential benefits of increasing canopy cover could be offset by increased stem and root respiration. While we did not undertake a full life cycle assessment of the C fluxes associated with mulch or the C emissions associated with lawn or tree maintenance [18, 19], the Scope 1 implications are clear nonetheless. Past studies [18, 42, 43] show that both lawn mowing and fertilizer use represent hidden C costs that reduce the overall C sequestration capacity of lawns. Generally, lawns are net C negative (resulting in additional C release to the atmosphere), though reducing C-intensive landscaping techniques would facilitate greater C sequestration potential.

While large-scale reductions of Boston University’s fossil fuel emissions are central to achieving net C neutrality, biogenic fluxes represent an important component of the greenhouse gas budget. Land owners such as universities should consider their biogenic emissions and the role of their management choices in both storing C and creating large Scope 1 sources through import and release of C through mulch, and the potential for uptake through canopy expansion.

As global climate policies slowly advance, it has become increasingly important for independent institutions to contribute to reductions in C emissions to achieve the established goal of limiting global temperature rise to 2 °C or less above pre-industrial levels [44]. Although Boston University has pledged to reduce fossil fuel emissions through a series of offsets and renewable energies, the institution should ensure that local C fluxes are also accounted for in its University-wide C quantification. While biogenic C fluxes represent a small proportion of the total C emissions from Boston University, its urban campuses cannot achieve true net C neutrality without first considering biogenic C fluxes. Further, net biogenic C neutrality is more readily feasible if the University adopts revised approaches to beautification of the campus through landcover management.

## Conclusions

Green infrastructure and nature-based solutions cannot be assumed to result in biogenic C sinks. We show that C fluxes from mulched and lawn landcover via soil respiration on Boston University’s three urban campuses outweigh C sequestration by on-campus vegetation, making each of the University’s urban campuses net biogenic C sources. Emissions via soil respiration were found to be significantly higher in mulch than lawn landcover per unit area, although on two campuses, lawn and mulch contributed roughly equal amounts of C to the overall CO_2_ flux to the atmosphere due to a larger proportion of lawn landcover. Additionally, we show that although anthropogenic emissions are considerably larger than biogenic emissions, biogenic fluxes into grass and woody biomass still have the potential to offset a portion of anthropogenic emissions while providing additional ecosystem services. Finally, we conclude that biogenic C emissions from urban university campuses can be reduced through reduction in the frequency and quantity of mulch addition onto urban campuses and through increasing the number of trees.

## Supplementary information

**Additional file 1.** Landcover on Boston University’s urban campuses in the City of Boston overlaid on high-resolution aerial imagery from MassGIS (https://docs.digital.mass.gov/dataset/massgis-data-usgs-color-ortho-imagery-2019). The tree canopy layer has been made transparent to allow for visualization of layered landcovers when zoomed in.

## Data Availability

The data is published on the Harvard Dataverse at https://dataverse.harvard.edu/dataset.xhtml?persistentId=doi%3A10.7910%2FDVN%2FRJSGG0.

## Abbreviations

C: Carbon
CO_2_: Carbon dioxide
PVC: Polyvinyl chloride
LOESS: Locally estimated scatterplot smoothing
LiDAR: Light detection and ranging
*bm*: Biomass
DBH: Diameter at breast height
ACES: Anthropogenic carbon emissions system
MTCO_2_e: Metric tons of carbon dioxide equivalent
NEIDL: National emerging infectious diseases laboratories
