# Supplementary figures and images for: Influence of landscape management practices on urban greenhouse gas budgets
Source: Carbon Balance Manag. 2021 Jan 7;16:1. doi: 10.1186/s13021-020-00160-5 (PMC7792215; doi:10.1186/s13021-020-00160-5)

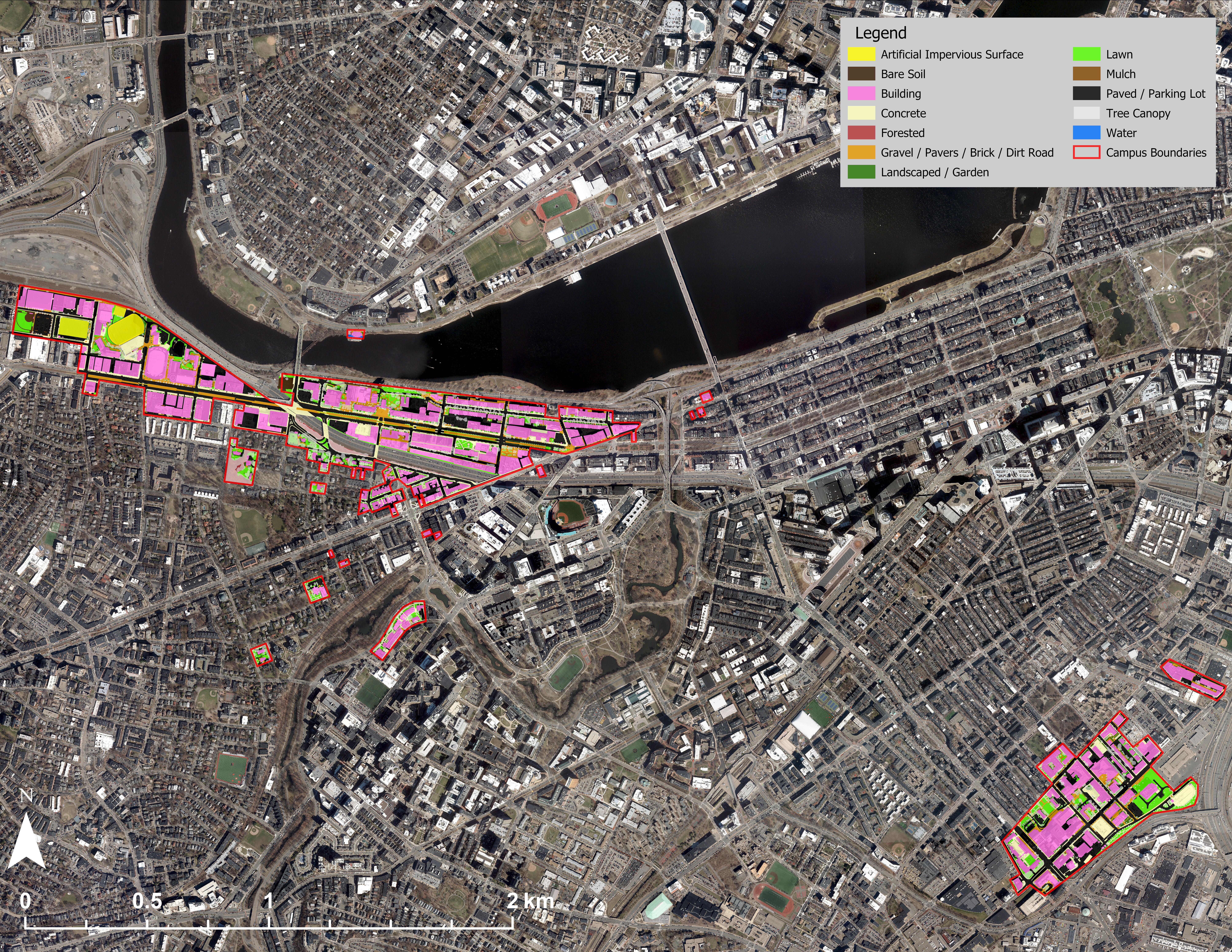

Supplement: Supplementary file 1 — Additional file 1. Landcover on Boston University’s urban campuses in the City of Boston overlaid on high-resolution aerial imagery from MassGIS (https://docs.digital.mass.gov/dataset/massgis-data-usgs-color-ortho-imagery-2019). The tree canopy layer has been made transparent to allow for visualization of layered landcovers when zoomed in. [file 13021_2020_160_MOESM1_ESM.jpg]
